# Serum Markers Associated with Disease Severity in a Bosnian Hemorrhagic Fever with Renal Syndrome Cohort

**DOI:** 10.3390/v14071377

**Published:** 2022-06-24

**Authors:** Danny Noack, Maja Travar, Visnja Mrdjen, Jolanda J. C. Voermans, David van de Vijver, Richard Molenkamp, Marion P. G. Koopmans, Marco Goeijenbier, Barry Rockx

**Affiliations:** 1 Department of Viroscience, Erasmus University Medical Center, 3015 GD Rotterdam, The Netherlands; d.noack@erasmusmc.nl (D.N.); j.voermans@erasmusmc.nl (J.J.C.V.); d.vandevijver@erasmusmc.nl (D.v.d.V.); r.molenkamp@erasmusmc.nl (R.M.); m.koopmans@erasmusmc.nl (M.P.G.K.); m.goeijenbier@erasmusmc.nl (M.G.); 2 Department for Clinical Microbiology, University Clinical Center of Republika Srpska, 78000 Banja Luka, Bosnia and Herzegovina; maja.travar@kc-bl.com (M.T.); visnja.mrdjen@kc-bl.com (V.M.); 3 Department for Microbiology and Immunology, Faculty of Medicine, University of Banja Luka, 78000 Banja Luka, Republika Srpska, Bosnia and Herzegovina; 4 Department of Intensive Care, Erasmus University Medical Center, 3015 GD Rotterdam, The Netherlands

**Keywords:** orthohantavirus, Puumala virus, hemorrhagic fever with renal syndrome, nephropathia epidemica, patient cohort, serum markers, virus neutralization

## Abstract

Puumala orthohantavirus (PUUV) is endemic in Europe and can cause hemorrhagic fever with renal syndrome (*nephropathia epidemica*). Disease features include fever, thrombocytopenia, and acute kidney injury (AKI). This retrospective cohort study of forty PUUV patients aims to characterize associations of serum immunological, hemostatic or kidney injury markers to disease severity. While interleukin-18 (IL-18) was significantly increased in severely thrombocytopenic patients (<100 × 10^9^ platelets/L) compared to patients with higher platelet counts, RANTES was significantly decreased in these patients. These data suggest that patients with significant thrombocytopenia might have experienced pronounced Th1 immune responses. When kidney dysfunction was used as the primary disease outcome, recently identified AKI biomarkers (Cystatin C, insulin-like growth factor-binding protein 7, Nephrin, and trefoil factor 3) were significantly upregulated in patients with severe PUUV infection, defined as the estimated glomerular filtration rate (eGFR) below 30 m/min/1.73 m^2^. The increased expression of these markers specifically indicates pathology in glomeruli and proximal tubuli. Furthermore, E-selectin was significantly higher while interferon gamma-induced protein 10 (IP-10) was significantly lower in PUUV patients with more severe kidney dysfunction compared to patients with higher eGFR-values. Increased E-selectin illustrates the central role of endothelial cell activation, whereas decreased IP-10 could indicate a less important role of this cytokine in the pathogenesis of kidney dysfunction.

## 1. Introduction

Orthohantaviruses are zoonotic viruses able to cause disease in humans. These viruses generally exist in virus-specific reservoir rodent species spread across the globe, and the incidence of infections in humans is driven by the dynamics of infection in the reservoir host population, leading to seasonal and multi-annual fluctuations and sporadic local outbreaks [1]. Bosnia-Herzegovina is considered one of Europe’s hotspots for orthohantaviruses. In the general human population in endemic regions, the seroprevalence for the most detected orthohantavirus, Puumala virus (PUUV), is estimated to be as high as 6% [2]. During the period from 1 January 2017 until 30 September 2017, a total of 128 patients that had positive serology for orthohantaviruses were registered in Bosnia-Herzegovina. The center of the outbreak was located in the northwestern part of Bosnia-Herzegovina, where 95 infected persons were recorded (Republic of Srpska entity). The first case was recorded on 5 March 2017. The majority of the cases occurred in July and August, whereas the last recorded case that year was on 30 September. The majority of the cases were in the northwestern part of the country in the municipalities of Gradiska, Kostajnica, Novi Grad, and Kozarska Dubica, in places near the rivers Sava and Una. PUUV is considered the most prevalent orthohantavirus in Europe and can spread from infected bank voles (*Myodes glareolus*) to humans through the virus containing aerosolized excreta. Symptomatic PUUV infection results in a mild form of hemorrhagic fever with renal syndrome (HFRS), known as *nephropathia epidemica* (NE). The hallmark feature of NE is proteinuria with subsequent acute kidney injury (AKI), in certain cases even requiring hemodialysis. The most common symptoms include fever, general malaise, myalgia, gastro-intestinal discomfort, blurred vision, and oliguria in the acute phase [3,4]. In the center of this outbreak, the majority of the hospitalized patients had mild clinical presentation, with no lethal outcomes. The pathogenesis of PUUV is believed to be mostly driven by increased vascular permeability, thrombocytopenia, and an increased immune response as a consequence of endothelial cell infection in different organs [5]. Accordingly, previous PUUV patient cohort studies have looked into biochemical disease markers concerning immunological, hemostatic or kidney injury markers [6,7,8]. However, none of these studies specifically combined markers for all three pillars of pathogenesis in one study. Patients suffering from more coagulation-related symptoms might undergo a different disease course to develop these clinical outcomes in a specific moment in time compared to patients with more kidney-related issues. Improving our understanding of the NE disease course will aid in more personalized clinical approaches in treating these patients. Therefore, we characterized potential biomarkers for disease severity in a cohort of PUUV patients from an outbreak in Bosnia-Herzegovina.

## 2. Materials and Methods

### 2.1. Study Population and Ethics

A large outbreak of HFRS in Republika Srpska (part of Bosnia and Herzegovina) took place outside and inside endemic regions in late spring and summer in 2017. Anonymized demographic, clinical, and laboratory data of the patients hospitalized with HFRS in University Clinical Center of Republika Srpska from 1 May 2017 until 30 September 2017 were extracted from the hospital database. This hospital is the largest and main hospital in the Republic of Srpska, where patients from all over the country were hospitalized. During the study period and beyond, this hospital has been a participant within the Multi-centre EuRopean study of MAjor Infectious Disease Syndromes (MERMAIDS) project. This study was covered by ethical clearance provided by MERMAIDS. The study protocol is available on the website of the Platform foR European Preparedness Against (Re-)emerging Epidemics (PREPARE): https://prepare.ersnet.org/trials-protocols.aspx, accessed on 9 February 2021.

This study was approved by the University of Oxford (reference number MERMAIDS ARBO, OXTREC 31-15) and the Ethical Board of the University Clinical Center of the Republic of Srpska (document number 01-9-119.2/16 on 23 March 2016).

### 2.2. Sample Selection

Blood samples from patients were collected at a median of six days (range 3–10) post-symptom onset. Routine serological diagnostics were performed by a diagnostic kit for anti-hantavirus pool Eurasia ELISA IgM and IgG antibodies against Hantaan orthohantavirus, Dobrava-Belgrade orthohantavirus (DOBV) or PUUV (EUROIMMUN AG, Lübeck, Germany) at hospital admission in Bosnia-Herzegovina. From 95 patients who tested positive for IgM and IgG antibodies, we selected samples from 40 patients hospitalized during different periods of the outbreak (May, June, July, August, September 2017) and from different parts of the country (municipalities of the towns Gradiska, Kozarska Dubica, Kostajnica, Banja Luka, Bijeljina, Doboj, Trebinje). Selected serum aliquots were sent to Erasmus MC, Rotterdam, the Netherlands, where molecular and serological assays were performed, confirming PUUV infection. Aliquots were stored at −80 °C until diagnostic assays or multiplex immunoanalyses.

### 2.3. Retrospective Diagnostics

Serum samples were tested for RNA-emia by quantitative reverse transcription polymerase chain reaction (RT-qPCR) and for PUUV-specific antibodies by an in-house developed micro-neutralization test (MNT), loosely based on [9]. First, patient sera were tested for the presence of PUUV RNA. RNA extraction of these samples was performed via Magnapure LC with a Total Nucleic Acid Isolation kit (Roche, Mannheim, Germany) or manually with a High Pure RNA Isolation Kit (Roche, Mannheim, Germany) according to the manufacturer’s instructions. PUUV RNA presence was tested by amplification via an in-house-designed Taqman primer-probe set Puumala_fwd_4 ATGTGAAACTGAGCTATCCC, Puumala_fwd4b ATGTGAAACTGAGCTATCCT, Puumala_rev AGTAGTAGACTCCTTGAAAAGC and Puumala_probe AGCATATATATAAG-6FAM-TACACAAYWTACTACCTCAACATGCTGA-BHQ-1 with real-time PCR Taqman Fast Virus 1-step master mix (ThermoFisher Scientific, Waltham, MA, USA) for 5 min at 50 °C, 20 s at 95 °C, and 45 cycles (3 s at 95 °C, 30 s at 60 °C) on a LC480-system (Roche, Mannheim, Germany) or a 7500 Real-Time PCR System (Applied Biosystems, Waltham, MA, USA). As viral loads are usually already declining at the time that more severe symptoms present themselves, an alternative method is required to identify the causative orthohantavirus species [10]. Concurrently, antibody responses are mounted with IgM and IgG antibodies present in patient sera that can be used for this purpose. Therefore, all sera were serotyped by comparing virus neutralization capacities for PUUV and DOBV, the two most prevalent orthohantaviruses in Bosnia-Herzegovina [4]. In short, heat-inactivated serum dilutions (starting 1:50) were co-incubated with 100 TCID_50_ virus per well for two hours, after which this serum–virus mix was used to inoculate Vero E6 cells (ATCC, Manassas, VA, USA) in triplicate on 96-well plates. Viruses used were either PUUV (EVAg no. 008V-EVA1472, P + 3) or DOBV (EVAg no. 008V-03724, P + 2) in Dulbecco’s Modified Eagle Medium (Lonza, Walkersville, MD, USA) supplemented with 10% fetal calf serum, HEPES, sodium bicarbonate, and 1% penicillin–streptomycin (Lonza, Walkersville, MD, USA) at 37 °C in a humidified CO_2_ incubator. All cells and virus stocks were confirmed to be free of mycoplasma. Post-incubation, cells were fixed with ice-cold absolute ethanol and stained for immunofluorescence by using rabbit anti-PUUV nucleoprotein serum/Ig (1:500, NR-9675, BEI Resources, NIAID, NIH, Manassas, VA, USA) or rabbit anti-DOBV nucleoprotein serum/Ig (1:500, NR-12152, BEI Resources, NIAID, NIH, Manassas, VA, USA) followed by polyclonal goat anti-rabbit Alexa Fluor 488-conjugated antibodies (1:1000, Invitrogen, Eugene, OR, USA). PUUV diagnosis was confirmed by a positive signal (Ct value < 40) in RT-qPCR. If RT-qPCR was negative, the following were used as criteria for PUUV diagnosis: the presence of MNT titers for PUUV with none for DOBV or at least four-fold higher MNT titers for PUUV, thereby excluding extensive cross-reactivity with DOBV. Of note, due to limited sample availability, serum 626 was not tested at higher dilutions during MNT. As part of clinical data analyses, kidney function was evaluated by calculating the estimated glomerular filtration rate (eGFR) based on the serum creatinine, age, and sex of each patient [11]. 

### 2.4. Multiplex Immunoassays

To determine endothelial cell activation and coagulation markers, a human thrombosis panel (10-plex) kit (LEGENDplex, BioLegend, San Diego, CA, USA) was used according to the manufacturer’s instructions. Measured proteins included D-dimer, factor IX, interleukin-6 (IL-6), IL-8, plasminogen activator inhibitor-1 (PAI-1), P-selectin, P-selectin glycoprotein ligand-1 (PSGL-1), soluble CD40 ligand (sCD40L), tissue plasminogen activator (tPA), and tissue factor (TF). Patient sera were diluted 1:50 prior to measurements. The LEGENDplex Data Analysis Software Suite was used to analyze LEGENDplex flow cytometry data files. Furthermore, two custom Magnetic Luminex assays (R&D Systems, Minneapolis, MN, USA) were designed for measuring known AKI biomarkers and additional endothelial cell activation markers, i.e., RANTES, Cystatin C, galectin-3 binding protein (Galectin-3BP), neutrophil gelatinase-associated lipocalin (NGAL) (1:50 sera dilution), and E-selectin, intercellular adhesion molecule 1 (ICAM-1), urokinase plasminogen activator surface receptor (uPAR), vascular endothelial growth factor (VEGF), insulin-like growth factor-binding protein 7 (IGFBP-7), IL-18, interferon gamma-induced protein 10 (IP-10), kidney injury molecule-1 (KIM-1), Nephrin, Osteopontin, pentraxin 3, and trefoil factor 3 (TFF3) (1:2 sera dilution). Samples with concentrations below the detection limit were assigned the value of the lowest detection limit for further analyses. As expected for sera, TF concentrations were all below the threshold and therefore excluded from analyses.

### 2.5. Statistical Analyses

Serum marker comparisons between severely and milder diseased patient groups (grouping based on blood platelet levels and eGFR values) and timing of sampling post-symptom onset were compared by the Mann–Whitney U test; *p*-values < 0.05 were considered statistically significant. Statistical analyses were performed in GraphPad Prism version 7. 

## 3. Results

### 3.1. Patient Cohort Characteristics

Forty patients were included in this study (Table 1). Combining RT-qPCR results, which were negative in most patients, with virus MNT titers of sera samples allowed for retrospective PUUV diagnosis of all forty patients (Table 2). Thirty patients (75%) demonstrated fever defined as ≥38.0 °C. Four patients (10%) required hemodialysis, of which two (5%) were transferred to the intensive care unit (ICU). All patients recovered completely.

### 3.2. Severe Thrombocytopenia

**Figure 1:**
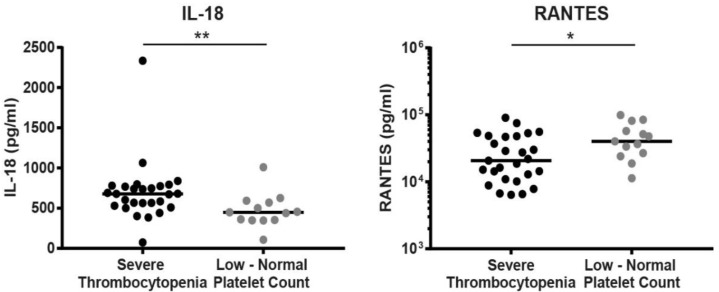
Serum markers correlated with severe thrombocytopenia in PUUV patients. Patients were considered to display significant thrombocytopenia when platelet counts were <100 × 10^9^/L. Statistical significance was calculated by the Mann–Whitney U test; *, indicating a *p*-value < 0.05, and **, indicating a *p*-value < 0.01, were considered statistically significant. Abbreviations: IL-18, interleukin-18.

In this cohort, only four patients (10%) did not demonstrate thrombocytopenia according to the conventional clinical definition (platelet count <150 × 10^9^/L). As platelet levels can be a crucial factor in following disease progression by clinicians, setting a more stringent definition of thrombocytopenia (i.e., severe thrombocytopenia) was essential for allowing proper grouping in this patient cohort. Therefore, in order to determine biomarkers that are associated with severe thrombocytopenia, patients were divided into two groups: patients with severe thrombocytopenia (<100 × 10^9^ platelets/L; *n* = 27) versus ones with low to normal platelet counts (≥100 × 10^9^ platelets/L; *n* = 13). Timing of sampling post-onset of symptoms was not significantly different between the severe thrombocytopenia group (median 6 days, range 3–10 days) and the less severe group (7, 5–10 days, *p* = 0.15) as determined by the Mann–Whitney U test. RANTES (CCL5) levels were significantly decreased in patients, demonstrating severe thrombocytopenia (median 20,767 pg/mL compared to 40,056 pg/mL), while IL-18 levels were significantly increased in these patients (Figure 1; 677.9 vs. 448.1 pg/mL). Other serum markers were not significantly different in patients with severe thrombocytopenia (Table 3). Of these twenty-seven patients, eleven (41%) presented with clinically observed hemorrhages. In the other group with higher platelet counts, five patients (39%) demonstrated hemorrhages, showing that clinically observed hemorrhages could not be used as a substitute for the severity degree of thrombocytopenia with the current definition of severe thrombocytopenia.

### 3.3. Acute Kidney Injury

**Figure 2:**
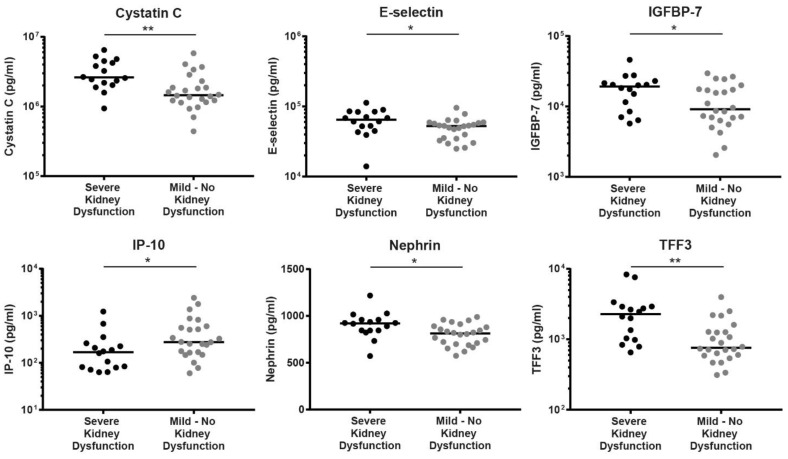
Serum markers correlated with severe kidney dysfunction in PUUV patients. Patients with severe kidney dysfunction were identified by eGFR-values <30 m/min/1.73 m^2^. Statistical significance was determined by the Mann–Whitney U test; *, indicating a *p*-value <0.05, and **, indicating a *p*-value < 0.01, were considered statistically significant. Abbreviations: IGFBP-7, insulin-like growth factor-binding protein 7; IP-10, interferon gamma-induced protein 10; TFF3, trefoil factor 3.

Since AKI is the hallmark symptom of NE, better understanding of which pathological factors contribute to disease progression into more severe kidney injury is of great interest. In order to identify biomarkers for kidney dysfunction, expressed as a decreased glomerular filtration rate (eGFR), patients were grouped as suffering from severe kidney dysfunction (eGFR < 30 m/min/1.73 m^2^) or mild to no kidney dysfunction (eGFR ≥ 30 m/min/1.73 m^2^). Timing of sampling post-onset of symptoms was only slightly different between the severe kidney dysfunction group (median 7.5 days, range 3–10 days) and the less severe group (6, 3–9 days, *p* = 0.04). For patients with severe kidney dysfunction, kidney toxicity markers Cystatin C (Cystatin 3; 2,613,100 vs. 1,454,550 pg/mL), IGFBP-7 (19,131 vs. 9114 pg/mL), Nephrin (921 vs. 813.5 pg/mL), and TFF3 (2281 vs. 760.5 pg/mL) were significantly increased (Figure 2). Further, E-selectin was significantly higher (64,632 vs. 52,554 pg/mL) and IP-10 significantly lower (CXCL10; 168.3 vs. 276.5 pg/mL) in patients with decreased eGFR (Figure 2). Other serum markers did not significantly differ between the two groups (Table 4). As anticipated, all patients requiring hemodialysis or intensive care treatment were present in the group with severe kidney dysfunction.

## 4. Discussion

PUUV infection is associated with thrombocytopenia and acute kidney injury. This retrospective PUUV patient cohort study aimed to identify biomarkers associated with more severe disease in terms of platelet count and kidney injury. IL-18 is an important pro-inflammatory cytokine often found to be upregulated in PUUV patients compared to healthy controls, indicating a role in pathogenesis [12,13,14,15]. In the current study, IL-18 was significantly higher in patients in the low platelet group. Previously, IL-18 was found to be decreased in the sera of HFRS patients, while being increased during the often more severe hantavirus cardiopulmonary syndrome (HCPS) [6]. A more prominent Th1-type immune response (lead by IL-18) was hypothesized to occur in HCPS compared to HFRS. This hypothesis could be translated to the more severe platelet reductions observed in PUUV patients, suggesting a more pronounced cytotoxic response. In agreement with this hypothesis, RANTES, which is normally upregulated late after T-cell activation, was indeed significantly decreased in severe thrombocytopenic patients and in late HFRS patients [6]. Similar mechanisms are also observed during other virus infections associated with thrombocytopenia [16]. Previous studies indicated that high plasma IL-6 and pentraxin 3 were associated with low platelet counts and clinical severity of NE [17,18,19]. One of the studies demonstrated a correlation between maximum pentraxin 3 and IL-6 levels in patient plasma [18]. Pentraxin 3 production is dependent on pro-inflammatory signals, such as IL-6. As IL-6 levels were below the detection limit for most patients in the current cohort, which might explain why no significant increases were observed for both factors in the current study.

In addition, significant increases of kidney injury markers could be detected in patients with lower eGFR. Plasma levels of Cystatin C have been suggested to be a more sensitive marker compared to classical measures such as creatinine and urinary albumin in predicting kidney injury during PUUV infection [20]. In accordance, in this cohort, patients with severe kidney dysfunction also demonstrated higher levels of serum Cystatin C. In addition to Cystatin C, serum IGFBP-7 levels were also increased in severely diseased patients compared to patients with better kidney function. In an ICU setting, urinary IGFBP-7 levels, especially when combined (i.e., multiplied) with urinary tissue inhibitor of metalloproteinases-2 (TIMP-2) levels, yield an excellent biomarker that positively correlates with the prediction of moderate to severe AKI [21]. As urinary samples were not measured in the current study, these findings could not be confirmed. Nevertheless, serum IGFBP-7 levels were already demonstrated to be significantly upregulated in severe PUUV patients. Moreover, significant increases in serum Nephrin were detected in patients with severe kidney dysfunction as expressed by decreased eGFR-values. A recent study showed a similar association, demonstrating significant increases in urinary Nephrin levels in PUUV patients with severe proteinuria compared to mild proteinuria [22]. However, a direct correlation between serum and urinary Nephrin levels during proteinuria is challenging to observe as shown in a study on pregnant women with severe preeclampsia [23]. These findings emphasize the importance of elucidating whether during PUUV-induced proteinuria urinary Nephrin originates from systemic circulation or solely from direct podocyte excretion. Finally, increased serum TFF3 levels were observed in patients with severe kidney dysfunction. A study focusing on identifying disease markers in urinary samples of PUUV patients also found increased TFF3 levels during early NE compared to healthy controls [7]. Altogether, PUUV patients with severe kidney dysfunction seem to display upregulated kidney injury markers indicating specifically glomerular (Cystatin C, Nephrin) and proximal tubular dysfunction (Cystatin C, IGFBP-7 and TFF3) [24,25]. Correspondingly, glomerular and proximal tubular cells are also the PUUV infection sites within the kidneys [26,27]. Ultimately, endothelial cell activation and immunopathogenesis play an important role in pathogenesis. This aspect is highlighted by the significant upregulation of endothelial inflammation marker E-selectin in severe PUUV cases compared to milder cases or in overall PUUV cases compared to HFRS cases caused by DOBV [8]. This study also reported that serum IP-10 levels were significantly higher in severe PUUV patients compared to those with mild disease [8]. However, in the current study, IP-10 levels were significantly decreased in severe patients compared to patients with less kidney dysfunction. The previous study combined thrombocytopenia and kidney dysfunction in the definition of severe disease, whereas the current study only demonstrated an association of decreased IP-10 levels in patients solely grouped based on severe kidney dysfunction. This may suggest that increased IP-10 plays a more prominent role in the development of severe thrombocytopenia compared to kidney dysfunction.

The current retrospective cohort study has limitations as no age- and gender-matched healthy controls or non-PUUV patients with similar clinical symptoms were included. Further, the relatively low total patient number (*n* = 40) did not allow for further statistical stratification, e.g., multivariate logistic regression analyses to identify independent markers associated with disease severity. Furthermore, an additional consequence of this is a skewness toward (young) men compared to women in this cohort. Consequently, future studies are required to confirm that these identified markers correlate specifically with PUUV-induced thrombocytopenia and AKI, which could play a role in the triage of patients for in- or outpatient treatment. Additionally, it would be of great interest to include other recently discovered blood markers that show the potential of being associated with severe disease, such as complement activation and glycoprotein YKL-40 [28,29,30]. Altogether, these findings demonstrate that specific biomarkers of interest in light of PUUV pathogenesis significantly differ between PUUV patients with different disease severity in terms of platelet counts and kidney injury. Future prospective cohort studies with sufficient patients are necessary to identify causal correlations and biomarkers that can be used to follow disease progression in clinical settings.

## Figures and Tables

**Table 1:** Characteristics of forty patients included in this cohort study.

Clinical or Laboratory Variable	Median or Number	Range
Patients, no.	40	N/A
Sex, female/male, no.	6/34	N/A
Age, median (range)	42	(16–69)
Sample collection on days post-onset of symptoms, median (range)	6	(3-10)
Mortality, yes/no, no.	0/40	N/A
Thrombocyte levels (<100 × 10^9^/L), median (range)	73.5	(39–435)
Clinically reported bleeding, yes/no, no.	16/14	N/A
Hemodialysis, yes/no, no.	4/36	N/A
Intensive care unit, yes/no, no.	2/38	N/A
Minimal estimated glomerular filtration rate (m/min/1.73 m^2^), median (range)	49	(5–136)
Urine output, anuric first day/oliguric/regular, no.	4/31/5	N/A
Pulse at admission, median (range)	67.5	(60–90)
Mean arterial pressure, median (range)	96.7	(70–173.3)
Reported diabetes mellitus 2, yes/no, no.	3/37	N/A
Reported hypertension, yes/no, no.	5/35	N/A

**Table 2:** Retrospective diagnostics of patient sera. Detectable Ct-values (<40) for PUUV RNA-confirmed PUUV diagnosis. In addition, the presence of PUUV-neutralizing titers determined during micro-neutralization test (MNT) with the absence of DOBV MNT titers or four-fold higher PUUV MNT titers also confirmed PUUV diagnosis. Serum 626 was not tested to higher dilution in MNT due to limited sample availability. Abbreviations: ID, identity; RT-qPCR, quantitative reverse transcription polymerase chain reaction; Ct-value, cycle threshold value; MNT, micro-neutralization test; PUUV, Puumala virus; DOBV, Dobrava-Belgrade virus.

Serum ID	RT-qPCR	Ct-value	MNT PUUV	MNT DOBV	Diagnosis	Serum ID	RT-qPCR	Ct-Value	MNT PUUV	MNT DOBV	Diagnosis
274	-	-	1600	<50	PUUV	527	PUUV	33.8	400	<50	PUUV
334	-	-	800	100	PUUV	530	-	-	400	<50	PUUV
338	PUUV	34.9	3200	<50	PUUV	536	PUUV	36.7	800	50	PUUV
357	PUUV	36.9	800	50	PUUV	542	PUUV	35.4	800	<50	PUUV
365	-	-	6400	<50	PUUV	556	-	-	800	<50	PUUV
368	-	-	400	<50	PUUV	559	-	-	200	<50	PUUV
375	-	-	400	<50	PUUV	562	PUUV	38.6	800	<50	PUUV
393	-	-	400	<50	PUUV	563	-	-	800	<50	PUUV
394	-	-	800	<50	PUUV	570	-	-	6400	<50	PUUV
395	-	-	3200	50	PUUV	579	-	-	800	<50	PUUV
405	-	-	1600	<50	PUUV	581	PUUV	36.5	50	<50	PUUV
413	-	-	800	<50	PUUV	582	-	-	100	<50	PUUV
425	-	-	400	100	PUUV	583	-	-	200	<50	PUUV
497	-	-	400	<50	PUUV	586	PUUV	37.0	400	<50	PUUV
498	-	-	400	<50	PUUV	588	PUUV	37.8	100	<50	PUUV
504	PUUV	38.0	400	<50	PUUV	619	-	-	100	<50	PUUV
505	PUUV	35.4	800	<50	PUUV	622	PUUV	35.0	400	<50	PUUV
507	-	-	1600	<50	PUUV	626	PUUV	33.5	≥3200	≥3200	PUUV
517	PUUV	36.9	400	<50	PUUV	627	PUUV	37.0	1600	400	PUUV
524	PUUV	35.7	6400	<50	PUUV	5737	-	-	1600	<50	PUUV

**Table 3:** Overview of serum marker levels in PUUV patients. Patients were considered to display severe thrombocytopenia when platelet levels were <100 × 10^9^/L. Statistical significance was calculated by the Mann–Whitney U test; *p*-values < 0.05 were considered statistically significant. Median (range). Abbreviations: ICAM-1, intercellular adhesion molecule 1; IL-6, interleukin-6; IL-8, interleukin-8; PAI-1, plasminogen activator inhibitor-1; PSGL-1, P-selectin glycoprotein ligand-1; sCD40L, soluble CD40 ligand; tPA, tissue plasminogen activator; uPAR, urokinase plasminogen activator surface receptor; VEGF, vascular endothelial growth factor; Galectin-3BP, galectin-3 binding protein; IGFBP-7, insulin-like growth factor-binding protein 7; IL-18, interleukin-18; IP-10, interferon gamma-induced protein 10; KIM-1, kidney injury molecule-1; NGAL, neutrophil gelatinase-associated lipocalin; TFF3, trefoil factor 3.

Serum Marker	Severe Thrombocytopenia (*n* = 27)	Low—Normal Platelet Count (*n* = 13)	*p*-Value
D-dimer (pg/mL)	1,463,837 (315,636–7,962,830)	585,048 (260,203–6,029,023)	0.0599
E-selectin (pg/mL)	52,904 (13,972–112,748)	61,120 (30,304–95,610)	0.2175
Factor IX (pg/mL)	1,126,515 (123,805–2,646,300)	1,230,664 (393,903–2,488,834)	0.9093
ICAM-1 (pg/mL)	410,492 (53,625–1,451,000)	241,146 (90,676–868,792)	0.5490
IL-6 (pg/mL)	104.6 (104.6–2487)	104.6 (104.6–1230)	0.9307
IL-8 (pg/mL)	35,876 (35,876–4,703,235)	35,876 (35,876–1,341,001)	0.3395
PAI-1 (pg/mL)	636,183 (233,006–1,870,126)	537,770 (264,323–3,269,881)	0.8867
P-selectin (pg/mL)	40,365 (9156–333,392)	29,217 (15,141–124,363)	0.9773
PSGL-1 (pg/mL)	48,518 (27,093–72,048)	39,528 (22,163–71,841)	0.1271
RANTES (pg/mL)	20,767 (6398–90,191)	40,056 (11,293-99,276)	0.0290
sCD40L (pg/mL)	43,816 (860.0–972,480)	93,321 (860.0–404,848)	0.3619
tPA (pg/mL)	43,589 (256.0–132,727)	22,108 (905.0–130,382)	0.3165
uPAR (pg/mL)	1386 (719.0–2727)	1238 (794.6–2034)	0.5927
VEGF (pg/mL)	97.18 (8.88–374.7)	88.29 (8.88–489.9)	0.4449
Cystatin C (pg/mL)	1,818,000 (702,466–5,819,300)	2,191,400 (440,681–6,472,400)	0.4539
Galectin-3BP (pg/mL)	1,378,500 (707,253–4,978,800)	1,259,500 (877,266–2,124,200)	0.3912
IGFBP-7 (pg/mL)	11,022 (2043–29,575)	15,889 (5729–45,940)	0.3197
IL-18 (pg/mL)	677.9 (72.97–2334)	448.1 (107.4–1010)	0.0042
IP-10 (pg/mL)	274.7 (59.97–2416)	164.5 (63.31–1234)	0.0559
KIM-1 (pg/mL)	103.5 (54.41–167.6)	111.1 (83.43–145.3)	0.5140
Nephrin (pg/mL)	834.7 (572.0–1218)	891.1 (665.0–1027)	0.1610
NGAL (pg/mL)	129,637 (45,709–591,532)	103,241 (41,860–219,892)	0.7757
Osteopontin (pg/mL)	35,207 (8486–247,279)	51,577 (3620–754,860)	0.8146
Pentraxin 3 (pg/mL)	8562 (1913–41,062)	6295 (2579–29,075)	0.1679
TFF3 (pg/mL)	1251 (336.6–7611)	982.5 (312.4–8333)	0.9319

**Table 4:** Overview of serum marker levels in PUUV patients. Patients were considered to display severe kidney dysfunction when eGFR-values were <30 m/min/1.73 m^2^. Statistical significance was calculated by the Mann–Whitney U test; *p*-values < 0.05 were considered statistically significant. Median (range). Abbreviations: ICAM-1, intercellular adhesion molecule 1; IL-6, interleukin-6; IL-8, interleukin-8; PAI-1, plasminogen activator inhibitor-1; PSGL-1, P-selectin glycoprotein ligand-1; sCD40L, soluble CD40 ligand; tPA, tissue plasminogen activator; uPAR, urokinase plasminogen activator surface receptor; VEGF, vascular endothelial growth factor; Galectin-3BP, galectin-3 binding protein; IGFBP-7, insulin-like growth factor-binding protein 7; IL-18, interleukin-18; IP-10, interferon gamma-induced protein 10; KIM-1, kidney injury molecule-1; NGAL, neutrophil gelatinase-associated lipocalin; TFF3, trefoil factor 3.

Serum Marker	Severe Kidney Dysfunction (*n* = 16)	Mild—No Kidney Dysfunction (*n* = 24)	*p*-Value
D-dimer (pg/mL)	614,979 (260,203–6,029,023)	1,280,942 (282,273–7,962,830)	0.8379
E-selectin (pg/mL)	64,632 (13,972–112,748)	52,554 (24,964–95,610)	0.0360
Factor IX (pg/mL)	1,178,590 (393,903–2,646,300)	1,087,530 (123,805–2,488,834)	0.8166
ICAM-1 (pg/mL)	420,550 (104,141–1,154,500)	288,051 (53,625–1,451,000)	0.3175
IL-6 (pg/mL)	104.6 (104.6–255.1)	104.6 (104.6–2487)	0.3349
IL-8 (pg/mL)	35,876 (35,876–1,341,001)	35,876 (35,876–4,703,235)	0.4344
PAI-1 (pg/mL)	608,817 (264,323–1,929,785)	567,400 (233,006–3,269,881)	0.7330
P-selectin (pg/mL)	59,708 (15,141–333,392)	29,164 (9156–126,873)	0.0702
PSGL-1 (pg/mL)	39,834 (22,163–72,048)	48,386 (27,093–69,005)	0.2790
RANTES (pg/mL)	38,531 (6398–84,374)	22,974 (6551–99,276)	0.2017
sCD40L (pg/mL)	69,466 (860.0–530,310)	67,983 (860.0–972,480)	0.8317
tPA (pg/mL)	30,254 (256.0–132,727)	41,553 (905.0–92,487)	0.3887
uPAR (pg/mL)	1390 (719.0–2727)	1306 (794.6–2439)	0.4730
VEGF (pg/mL)	152.7 (8.88–486.9)	70 (11.65–489.9)	0.1804
Cystatin C (pg/mL)	2,613,100 (938,353–6,472,400)	1,454,550 (440,681–5,819,300)	0.0015
Galectin-3BP (pg/mL)	1,458,250 (707,253–4,644,800)	1,324,500 (783,212–4,978,800)	0.6923
IGFBP-7 (pg/mL)	19,131 (5729–45,940)	9114 (2043–29,575)	0.0451
IL-18 (pg/mL)	478 (72.97–2334)	649.3 (348.5–1064)	0.1084
IP-10 (pg/mL)	168.3 (63.31–1234)	276.5 (59.97–2416)	0.0312
KIM-1 (pg/mL)	107.9 (54.41–145.3)	98.5 (80.68–167.6)	0.3184
Nephrin (pg/mL)	921 (572–1218)	813.5 (574.3–989.7)	0.0105
NGAL (pg/mL)	148,830 (77,732–591,532)	99,766 (41,860–328,496)	0.0747
Osteopontin (pg/mL)	45,389 (3620–754,860)	31,385 (5622–247,279)	0.7278
Pentraxin 3 (pg/mL)	9146 (2579–41,062)	7847 (1913–25,083)	0.9023
TFF3 (pg/mL)	2281 (652.8–8333)	760.5 (312.4–3967)	0.0011

## Data Availability

The data presented in this study are available on request from the corresponding author.
